# Ethnic Differences in Atypical Parkinsonism—is South Asian PSP Different?

**DOI:** 10.1002/mdc3.14182

**Published:** 2024-08-07

**Authors:** Bettina Balint, Shermyn Neo, Francesca Magrinelli, Eoin Mulroy, Anna Latorre, Maria Stamelou, Huw R. Morris, Amit Batla, Kailash P. Bhatia

**Affiliations:** ^1^ Department of Clinical and Movement Neurosciences UCL Queen Square Institute of Neurology London UK; ^2^ Department of Neurology University Hospital Zurich and University of Zurich Zurich Switzerland; ^3^ Department of Neurology National Neuroscience Institute Singapore Singapore; ^4^ Parkinson and Movement Disorders Department HYGEIA Hospital Athens Greece; ^5^ European University of Cyprus Nicosia Cyprus

**Keywords:** progressive supranuclear palsy, phenotype, genetics, neuroimaging, survival

## Abstract

**Background:**

Progressive supranuclear palsy (PSP) is a progressive atypical parkinsonian condition that results in severe disability. There are few studies of PSP in patients of non‐white European ancestry.

**Objectives:**

We aim to perform deep phenotyping in a South Asian PSP cohort to uncover possible ethnic differences in disease characteristics.

**Methods:**

Consecutive PSP patients had their clinical records reviewed for clinical features operationalized in the Movement Disorder Society (MDS)‐PSP diagnostic criteria and relevant investigations, including imaging and genetic tests. Clinical variables were summarized by descriptive statistics and Kaplan–Meier curves were generated for survival analysis.

**Results:**

Twenty‐seven patients, comprising Indians (78%), Pakistanis (11%) and Sri Lankans (11%) were included. Mean age of symptom onset was 63.8 ± 7.0 years and 22% of patients had an early age of onset (<60 years). The most common presenting symptom was parkinsonism (56%), followed by cognitive dysfunction (37%), falls (33%) and dysarthria (26%). The predominance types at final review were distributed across PSP‐RS (67%), PSP‐PGF (15%), PSP‐P (15%) and PSP‐F (4%). Atypical clinical features like cerebellar signs (33%), REM‐sleep behavior disorder (RBD) (55%), visual hallucinations (22%), and a family history of parkinsonism (20%) were evident in a proportion of patients.

**Conclusions:**

We present a South Asian cohort of PSP patients with a higher than previously reported percentages of early‐onset disease, family history and atypical clinical manifestations. These patients do not fit easily into the PSP phenotypes defined by the current MDS criteria. Dedicated clinicopathological and genetic tests are needed in this population to dissect the pathogenesis of clinically‐defined PSP.

Progressive supranuclear palsy (PSP) was first described in 1964 in a small, largely white European case series, as a neurodegenerative condition with a characteristic vertical supranuclear gaze palsy.^1^ Since then, PSP has emerged as a four‐repeat (4R) tauopathy, with different neuroanatomical deposition patterns corresponding to heterogeneous clinical phenotypes. This is reflected in the clinical diagnostic criteria, which describe different predominance types such as PSP with Richardson's Syndrome (PSP‐RS), PSP with parkinsonism (PSP‐P), PSP with progressive gait freezing (PSP‐PGF), PSP with corticobasal syndrome (PSP‐CBS), PSP with speech and language disorder (PSP‐SL) and PSP with frontal presentation (PSP‐F).^2^, ^3^ Early and accurate diagnosis is becoming more important in an era where a variety of new treatment approaches for tauopathies are being investigated. However, most of the PSP cohorts included in clinical trials, as well as studies to validate the new criteria by the International Parkinson and Movement Disorder Society (MDS),^4^, ^5^, ^6^, ^7^ are predominantly represented by populations of white European ancestry.^8^, ^9^, ^10^

Because of lack of information in other ethnicities, there is growing interest in studying Parkinson's disease (PD) and atypical parkinsonian conditions in these underrepresented populations. Establishing whether clinical presentations and underlying pathomechanisms of these conditions in diverse populations differ from Caucasians remains an unmet need. Rare studies of PSP in non‐white European populations have suggested there may be differences in presentation. For instance, a Japanese study reported a high frequency of 14% of PSP with cerebellar presentation (PSP‐C) amongst their PSP patients.^11^ Further reports of PSP‐C in East Asian countries,^12^, ^13^, ^14^, ^15^ as well as high rates of self‐reported REM‐sleep behavior disorder (RBD) and visual hallucinations in a multi‐ethnic South‐east Asian country^16^ support the notion that the clinical spectrum of PSP differs in individuals of European and Asian ancestry. To the best of our knowledge, the detailed clinical spectrum of PSP has not yet been investigated in South Asian patients.

## Methods

### Study Design

This was a single‐center retrospective case series of patients of South Asian descent with a PSP phenotype who attended outpatient movement disorder clinics at the National Hospital for Neurology and Neurosurgery in the United Kingdom (UK) between February 2012 and October 2023. Patients were recruited under ethics‐approved research protocol (UCLH: 04/N034). All videoed patients provided written consent for the recordings.

We searched our electronic health records for patients of South Asian descent carrying a diagnosis of parkinsonism, and patients were included if they met the inclusion criteria for probable PSP based on the MDS‐PSP criteria,^3^ as determined by movement disorders specialists (KB, BB, SN) through a chart review. The criteria were retrospectively applied for patients diagnosed prior to its publication. In order to examine the hypothesis of ethnicity‐related differences in the clinical presentation of PSP, we decided to include patients with certain exclusionary findings (visual hallucinations, appendicular ataxia, autonomic failure, positive family history of parkinsonism) who otherwise fulfilled the criteria. The Multiple Allocation eXtinction (MAX) rule was then used to derive the disease predominance type at final review for each patient.^17^ All available data including demographics clinical features, family history and investigations were evaluated. A cut‐off of 60 years was used to establish early‐onset PSP (EOPSP), based on two recent studies using thresholds of 55 and 65.^18^, ^19^

Clinical features were extracted from clinical records according to the operationalized definitions in the MDS‐PSP criteria. Additionally, other features frequently looked for in patients with atypical parkinsonism were also captured, including the presence of cerebellar signs, postural abnormalities (excluding retrocollis), evidence of RBD, visual hallucinations and dysautonomia (orthostatic hypotension, urinary retention). In cases where information regarding the clinical feature was not documented, it was presumed to be absent to avoid over‐estimating its prevalence, except for RBD which not routinely asked for in suspected cases of PSP until recently. Video recordings of neurological examination of individual patients where available, were reviewed for the relevant clinical signs. Formal neuropsychometric assessments were noted particularly for verbal fluency, bradyphrenia and dysexecutive syndrome.

Imaging consisted of magnetic resonance imaging (MRI) with T2* or SWI sequences and/or dopamine transporter scans. Genetic testing consisted of analysis of gene panels on whole‐exome or whole‐genome sequencing data matrix, whole mitochondrial genome sequencing, common mitochondrial DNA mutations and Southern blot or tethering PCR for repeat expansion disorders.

### Statistical Analysis

Clinical variables were summarized by descriptive statistics; mean and standard deviation were reported for continuous variables while frequencies and percentages were presented for categorical variables. Kaplan–Meier curves were generated and compared using logrank tests for survival analysis. All statistical analyses were performed using SPSS (IBM, version 26).

## Results

### Patient Demographics and Diagnosis

We identified 27 patients of South Asian descent over a 10‐year period, all who met criteria for probable PSP. Five patients were excluded due to insufficient clinical data/follow up, or eventual alternative diagnosis eg, Parkin‐related parkinsonism. The largest represented ethnicity was Indian (78%, 21/27), followed by Pakistani (11%, 3/27) and Sri Lankan (11%, 3/27). Of the patients from India, 11 were originally from Gujarat, with family names of Patel and Shah (6 and 3 patients, respectively, none who were related). Males formed 52% of the cohort (14/27) (Table 1). The mean age of symptom onset was 63.8 ± 7.0 years, and 22% of patients (6/27) had an early age of onset (<60 years). The majority of our patients (23/27) resided permanently in the UK for most of their adult lives.

**TABLE 1:** Demographics and clinical features of South Asian PSP patients (*n* = 27)*

Age at onset, mean (SD)	63.8 (7.0)
Young‐onset (<60 years), *n* (%)	6 (22)
Male, *n* (%)	14 (52)
Ethnicity, *n* (%)	
Indian	21 (78)
Pakistani	3 (11)
Sri Lankan	3 (11)
PSP predominance type, *n* (%)	
RS	18 (67)
PGF	4 (15)
P	4 (15)
F	1 (4)
Presenting symptoms, *n* (%)	
Parkinsonism	15 (56)
Falls	9 (33)
Cognitive dysfunction	10 (37)
Dysarthria	7 (26)
Cerebellar signs, *n* (%)	9 (33)
Nystagmus	4 (15)
Limb dysmetria	4 (15)***
Gait ataxia	1 (4)
Hypermetric saccades	1 (4)
Other features, *n* (%)	
REM sleep behavior disturbance; *n* = 20**	11 (55)
Visual hallucinations	6 (22)
Abnormal posture	3 (11)
Dysautonomia	2 (7)
Levodopa responsiveness, *n* (%)	7 (26)
Levodopa‐induced dyskinesia, *n* (%)	3 (11)
Family history of parkinsonism, n (%); *n* = 25**	5 (20)

Abbreviations: F: frontal lobe cognitive or behavioral presentations; P: parkinsonism; PGF: progressive gait freezing; PSP: progressive supranuclear palsy; REM: rapid eye movement; RS: Richardson's syndrome.

* Unless specified otherwise.

** Information available when specifically enquired about.

*** 1 patient had both nystagmus and limb dysmetria.

### Clinical Features

The most common presenting symptom was parkinsonism (56%, 15/27), followed by cognitive dysfunction (37%, 10/27), falls (33%, 9/27) and dysarthria (26%, 7/27). Of 15 patients with parkinsonism presentation, 5 had features of corticobasal syndrome, having additional limb apraxia (Videos 1 and 4). Some patients presented with more than one predominant symptom. The predominance types at final review with an average follow‐up duration of 7.0 ± 3.0 years were distributed as such: PSP‐RS (67%, 18/27), PSP‐PGF (15%, 4/27), PSP‐P (15%, 4/27) and PSP‐F (4%, 1/27).

**Video 1:** Typical progressive supranuclear palsy (PSP). Segment 1 shows a 61‐year‐old male of Indian (Gujarati) descent with classic PSP‐RS, who presented with gait imbalance and recurrent falls. The video taken 4 years after onset shows the typical features of hypomimia with a staring expression and frontalis overactivity. He exhibited blepharospasm when initiating eye movements. He had square‐wave jerks, vertical>horizontal supranuclear gaze palsy, dysarthria and severely impaired postural reflexes, requiring two person‐assistance to ambulate. He turned enbloc and had dystonic posturing in his right foot with a striatal toe. Segment 2 shows a 55‐year‐old Pakistani female with PSP‐P with corticobasal syndrome (CBS) overlap. This left‐handed woman presented with difficulty in writing and slowing of her gait. The video taken 3 years after onset shows hypomimia with frontalis overactivity, asymmetric (left > right) bradykinesia with corticobasal features of limb dystonia and apraxia on copying gestures in the left hand. Vertical saccadic slowing was better appreciated in a second video taken 2 years later. Her disease course was slightly atypical in its early age of onset and protracted duration of 14 years.

We observed cerebellar signs in 33% of our patients (9/27), comprising central nystagmus (4/27), limb dysmetria (4/27), gait ataxia (1/27) and hypermetric saccades (1/27) (Video 2). Where it was enquired, sleep disturbance compatible with RBD was noted in 55% (11/20). Visual hallucinations were reported in 22% (6/27) of patients and all had formed hallucinations (eg, deceased relatives, apparitions, powdered substance) that occurred in the 1st–6th year of disease, on a wide range of levodopa doses (0–900 mg/day). Abnormal postures, specifically Pisa syndrome and severe anterocollis, were noted in 11% (3/27) (Video 3) and dysautonomia in 7% (2/27). Other typical clinical features of PSP in our patients are detailed in the supplementary material (S1).

**Video 2:** Progressive supranuclear palsy (PSP) with cerebellar signs. Segment 1 shows an example of PSP‐PFG. This 70‐year‐old Indian male who presented with gait slowing which progressed to freezing within a year, developed cerebellar signs later in the disease course. The video taken 7 years after onset shows gait freezing at initiation, during straight walking and turning, which could be overcome with visual cues. Examination of the extraocular movements showed clear vertical saccadic slowing and gaze‐evoked horizontal nystagmus. Segment 2 shows a 63‐year‐old Gujarati female with PSP‐RS who had cerebellar signs at presentation. She initially experienced dysarthria, then rapidly developed recurrent falls and blurred vision. The video taken 2 years after onset shows typical PSP features of square‐wave jerks, vertical gaze palsy, eyelid opening apraxia, hypomimia with frontalis overactivity, and dysarthria; but in addition, she had bilateral upper limb dysmetria, worse on the right.

**Video 3:** Progressive supranuclear palsy (PSP) with abnormal postures. This 56‐year‐old Gujarati female had PSP‐P with features not commonly associated with the disease, including evidence of rapid eye movement (REM)‐sleep behavior disorder and significant abnormal postures. She had presented with slowing more on the left, but developed worsening postures and gait imbalance 3 years later. The videos taken 3–4 years after onset show classic eye signs of PSP (vertical saccadic slowing, round‐the‐house sign, square‐wave jerks, eyelid opening apraxia), a positive applause sign, asymmetric upper limb bradykinesia (left>right), impaired postural reflexes. The unusual feature demonstrated here is marked anterocollis and lateral trunk flexion.

Partial or good levodopa response was reported in 26% of patients (7/27), while 11% (3/27) developed levodopa‐induced dyskinesia, 2 in the limbs and 1 lingual (Video 4). Five of twenty‐five patients (20%) had at least one first‐degree relative with parkinsonism, with two affected relatives with a diagnosis of PSP.

**Video 4:** Progressive supranuclear palsy (PSP) with levodopa‐induced dyskinesias. Segment 1 shows a 79‐year‐old Indian male with PSP‐P with corticobasal syndrome (CBS) overlap who developed an unusual complication of levodopa therapy. He presented with slowing more on the left and noted abnormal posturing and inability to use his left arm soon after. He experienced recurrent fall 6 years later. The video taken 10 years after onset shows vertical gaze palsy and right hand bradykinesia and astereognosis. The left hand was held in a fixed dystonic posture. He had an interesting dyskinesia involving only the tongue, that occurred after levodopa escalation. Segment 2 shows a 76‐year‐old Gujarati patient with PSP‐RS who developed levodopa‐induced dyskinesias. She presented initially with difficulty with left hand use but suffered recurrent falls within 3 years. She experienced generalized dyskinesia 5 years later, while on low dose Sinemet 62.5 mg low dose levodopa (150 mg daily) [TDS]. The video taken 6 years after onset shows frontalis overactivity, apraxia of eyelid opening, vertical gaze palsy, asymmetric limb bradykinesia, dystonia and apraxia, worse on the left. Her dyskinesias, while generalized, were also worse on the left.

### Neuroimaging

Presynaptic dopaminergic imaging performed in 13 patients, was invariably abnormal. MRI was available for review in 26 patients, and midbrain atrophy with relative sparing of the pons was observed in 13 (50%). None of our patients had significant chronic microvascular changes. All 9 patients with cerebellar features underwent MRI brain. Cerebellar atrophy occurred in 33% (3/9) and pontocerebellar cistern enlargement was noted in 22% (2/9). Midbrain and superior cerebellar peduncle atrophy were observed in 44% (4/9) and 33% of patients (3/9), respectively. Additional findings of frontal, parietal and generalized atrophy were seen in 67% (6/9) (Table 2).

**TABLE 2:** Magnetic resonance imaging (MRI) findings of individual cases with cerebellar signs

ID	Cerebellar signs	Onset to scan (years)	Midbrain atrophy	SCP atrophy	Cerebellar atrophy	Pontocerebellar cistern enlargement	Others
1	Nystagmus	4	−	−	−	−	Mild frontoparietal atrophy
2	Dysmetria	1	+	+	+	−	−
3	Dysmetria	2	−	+	−	+	Mild frontoparietal atrophy
6	Nystagmus	n/a	−	−	−	−	Generalized atrophy
9	Nystagmus, dysmetria	2	−	−	+	+	Pontine atrophy, putaminal signal reduction
12	Gait	2	+	−	−	−	−
13	Dysmetria	5	+	−	+	−	Frontal atrophy
20	Hypermetric saccades	2	−	−	−	−	Frontal atrophy
24	Nystagmus	5	(+ after 8 years)	+	−	−	Generalized atrophy

Abbreviations: MRI: magnetic resonance imaging, SCP: superior cerebellar peduncle.

### Genetics and Other Investigations

Genetic tests were performed in a targeted fashion, based on clinical phenotype. Overall, 17 patients (Table S2) underwent genetic testing, all of which were negative. In the EOPSP patients, 4 of 6 had microtubule associated protein tau (MAPT) and non‐MAPT frontotemporal dementia‐related genes tested in a standard dementia panel. In patients with cerebellar signs, 6 of 9 were screened for the common spinocerebellar ataxia genes including *ATXN1* (SCA 1), *ATXN 2* (SCA2), *ATXN3* (SCA3) and *ATXN7* (SCA7). Other commonly tested genes included those for young‐onset Parkinson's disease, mitochondrial disorders, Huntington's disease and its lookalikes.

Following the recognition of a PSP phenotype in autoantibody‐related immune disorders,^20^, ^21^ patients seen in our clinics after 2018 were screened for these antibodies. Autoimmune antibodies to IgLON5, LGI1 and CASPR2 were negative in all six patients tested. Cerebrospinal fluid biomarkers of neurodegeneration, namely total‐tau, phosphorylated‐tau and β‐amyloid 1–42 (Aβ_42_), were negative for a concomitant Alzheimer's pathology in all 12 patients tested. Prominent cognitive presentations (in six patients) and corticobasal syndrome (in four patients) were the main indications for CSF analysis.

### Survival Analysis

**Figure 1:**
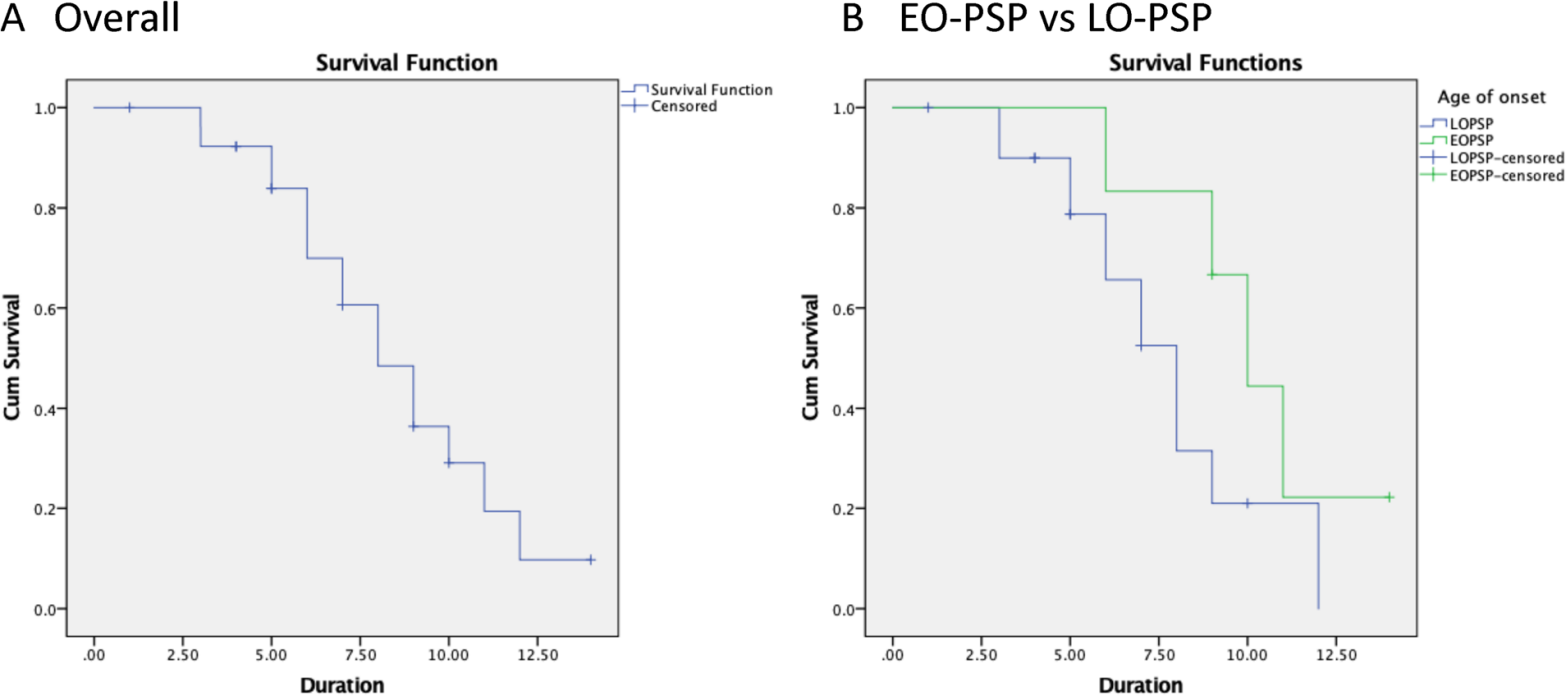
Survival analyses (Kaplan‐Meier curves) of patients with progressive supranuclear palsy (PSP) from symptom onset. (**A**) Overall survival probability (*y* axis) by years from symptom onset (*x* axis). Median survival was 8.0 years (95% CI: 6.2, 9.8). (**B**) Survival probability (*y* axis) by years from symptom onset (*x* axis) in early‐onset PSP (EOPSP) versus late‐onset PSP (LOPSP). Median survival for EOPSP was 10.0 years (95% CI: 8.0, 12.0) compared to 8.0 years (95% CI: 6.4, 9.6) in LOPSP (*P* = 0.137).

Of 27 patients, 16 were deceased, 8 alive and 3 lost to follow‐up. Overall, median survival from symptom onset to death was 8.0 years (95% CI: 6.2, 9.8) (Fig. 1a). There was a difference between survival times in patients with EOPSP (10.0; 95% CI: 8.0, 12.0) compared to those with late‐onset PSP (LOPSP) (8.0; 95% CI: 6.4, 9.6), but it did not reach statistical significant (*P* = 0.137) (Fig. 1b). There were no differences in survival times in patients with cerebellar signs or RBD compared to those without.

## Discussion

Our study, the first to specifically characterize PSP in patients of South Asian ancestry, found a distinctive clinical spectrum, namely that (1) a significant proportion of patients had early onset disease and/or a family history of parkinsonism, (2) parkinsonism was the most common initial clinical presentation, often with cortical signs, and (3) atypical features like cerebellar signs, RBD, visual hallucinations, postural deformities and levodopa‐induced dyskinesia were evident in a proportion of patients.

While the mean age of onset in our cohort (63.8 years) falls within the range of previously reported studies: 62–66 years,^22^, ^23^ we had a fair proportion of patients with early onset ie, age < 60 (22%, 6/27). This is higher than that reported in the PROSPECT study where 15% of their patients had symptom onset ≤59 years.^24^ Similar to their patients, the most common presenting symptom in our cohort (both EOPSP (3 of 6) and overall (15 of 27)), was parkinsonism. Although we did not find genetic diagnoses in our EOPSP patients, they had higher rates of family history of parkinsonism than the LOPSP patients (33% vs. 16%), thus raising the possibility of an undetermined genetic etiology or predisposition.

The commonest initial symptom presentation in our South Asian patients was parkinsonism. This differs from other PSP cohorts where postural instability with or without falls in the first 2 years of disease, was more prevalent.^23^, ^25^, ^26^ The majority of our patients (70%, 19/27) eventually developed recurrent falls within the first 3 years. Overall, cortical features at presentation appear to be more common in our cohort. Besides corticobasal syndrome (19%, 5/27), cognitive symptoms were noted at presentation in 37% of patients (10/27). Lower figures have been reported in other studies, possibly reflecting differences in phenotypic definition.^23^, ^24^, ^26^

The final predominance types were distributed across PSP‐RS (67%), PSP‐PGF (15%), PSP‐P (15%) and PSP‐F (4%), similar to previously described cohorts.^26^ Because our study only included patients with probable PSP, the SL, ocular motor dysfunction (OM) and postural instability (PI) predominance types were not represented.

Cerebellar signs were common in our patients (33%, 9/27). We ruled out common SCAs that may also present with parkinsonism in six patients where genetic testing was available. Cerebellar findings have also been described in other PSP cohorts—3 of 22 Japanese PSP patients in a clinicopathological study had cerebellar ataxia.^11^ This finding is not limited to Asian populations; an American study also reported that a significant proportion of patients (5 of 10) developed ataxia during the course of their disease.^27^ Involvement of the dentate nucleus in PSP was proposed as the cause of cerebellar signs. Indeed, Steele and colleagues described 4 of 9 patients who developed truncal and limb ataxia in their original paper.^1^

All patients with cerebellar signs had MRI, with the majority (5/9) performed within 1–2 years after symptom onset; 4 had evidence of cerebellar atrophy. Pontocerebellar cistern enlargement without widening of cerebellar fissures was observed in one patient, and has been described as a surrogate marker for cerebellar atrophy in PSP‐C.^13^ Of these 9 patients, 5 also demonstrated supportive imaging features of PSP—midbrain and/or superior cerebellar peduncle atrophy. Midbrain atrophy patterns have low rates of sensitivity, especially in early disease.^28^

A higher‐than‐expected proportion of our patients had features that are more commonly associated with synucleinopathies. In particular, 11 of 20 of our patients and their partners reported dream enactment suggesting RBD (this had to be recurrent and clinically convincing). The incidence of self‐reported RBD in other studies ranged from 0 to 23.5%,^16^, ^29^, ^30^ while REM sleep without atonia was observed in 20% of PSP patients.^29^ Additionally, 6 of 27 patients had formed visual hallucinations. Visual hallucinations are uncommon in studies of PSP, occurring in 2–5% of patients.^30^, ^31^ Formed hallucinations are associated with synucleinopathies and if present in the early stages of disease, suggest dementia with Lewy body (DLB) as a differential. However, a clinicopathological study evaluating the sensitivity and specificity of the MDS‐PSP diagnostic criteria found that patients with Lewy body disease never qualify for more than a diagnosis “suggestive of” PSP‐P.^6^ In another study of diffuse LDB, 11 out of 523 autopsy‐confirmed cases of DLDB had corticobasal syndrome clinically, with three demonstrating vertical gaze palsy.^32^ Interestingly, three patients in that study had DLDB and PSP co‐pathology, one with vertical gaze palsy.

Abnormal postures, Pisa syndrome and severe anterocollis, were observed in three of our patients. Pisa syndrome in PSP has been rarely reported in single cases^33^, ^34^ but a larger study of joint and skeletal deformities in patients with parkinsonism did not find any cases.^35^ Two patients had dysautonomia (orthostatic hypotension in one and urinary retention in both), but did not meet the diagnostic criteria for multiple system atrophy (MSA) because they had clear slowing of vertical saccades, an exclusion feature based on the latest MDS criteria.^36^ Dysautonomia can occasionally be seen in PSP.^37^

The high prevalence of a mixed phenotype in our cohort could be due to the presence of co‐pathology. A pathological study of 101 PSP patients showed that co‐pathology was the rule rather than the exception, occurring in 92%, with Alzheimer's disease (AD)‐related pathology being the most frequent.^38^ In our cohort, none of the 12 patients tested had CSF profiles consistent with AD. In another neuropathological study of parkinsonian disorders, 9% of PD cases had concomitant PSP pathology, while 20% of PSP cases had PD pathology.^39^ Cases of clinicopathological PSP with concomitant PD or DLB pathology have also been individually reported.^40^, ^41^ While concomitant pathologies may help us understand the overlaps between clinical and pathological diagnoses, there is probably a degree of clinical heterogeneity that exists within these disorders, resulting in PSP mimics. Notably, PSP‐parkinsonism, corticobasal syndrome and predominant frontal or presentation or cerebellar ataxia are more likely to have non‐PSP pathology.^2^ Richardson syndrome is most predictive of PSP neuropathology, although other 4R tauopathies occasionally lead to this phenotype. In a study evaluating the accuracy of the MDS‐PSP crtieria, 26 of 31 patients with clinically diagnosed PSP at their last visit had pathologically confirmed PSP while 5 had CBD.^6^ The majority of our patients had the RS subtype, thus we expect a lower likelihood of a misdiagnosis. A high incidence of cognitive symptoms, RBD, hallucinations and dysautonomia in PSP phenotypes has similarly been noted in Guadeloupean parkinsonism,^42^ in which neuropathology revealed a tauopathy identical or closely related to PSP.^43^ There is a lack of pathological studies in South Asian PSP patients and the question of co‐pathology/non‐PSP pathology in this ethnic group has yet to be answered.

The rate of clinical improvement with levodopa in our patients (26% (7/27) with partial or good response) was similar to current literature, with 20–30% of pathologically confirmed PSP patients reporting a beneficial response.^44^ Levodopa‐induced dyskinesias with unusual distribution were observed in 3 of 27 patients, 1 with little clinical benefit from levodopa. This was slightly more frequent that the reported prevalence rate of 4.4%.^45^ Dyskinesia in PSP typically have a facial distribution but patients with limb dyskinesias have been reported.^46^, ^47^ In an intriguing instance, one patient had combined Lewy body and PSP pathology.^46^

A family history of parkinsonism in first‐degree relations was present in 20% (5/25) of our patients, a rate higher than the 12% found in another study of familial aggregation of parkinsonism in PSP.^48^ Taken together with the evidence of higher rates of familial parkinsonism in EOPSP compared to LOPSP patients in our study as well as in previous literature,^24^ this finding suggests that genetic factors underlie this disorder. There was also a clustering of cases in Patel and Shah families in our cohort, possibly eluding to a genetic susceptibility in this subgroup. However, genetic screening in our study did not reveal any of the known MAPT or SCA mutations.

Finally, the median survival time of 8.0 years in our cohort was comparable to previous studies with disease durations ranging from 5 to 10 years.^49^ We found that the EOPSP patients had a different disease trajectory with a longer course (10.0 years), compared to LOPSP patients (8.0 years). This is supported by other studies that similarly found that a younger age of onset of symptoms was associated with a longer disease duration.^24^, ^25^ The most likely explanation for this is the presence of co‐pathology in LOPSP.^24^ Indeed, the proportion of patients with parkinsonism presentations were similar in both groups, suggesting that an over‐representation of PSP‐P predominance types in EOPSP was not the driving factor of the slower disease progression. The presence of cerebellar signs or RBD was not associated with survival times, consistent with previous literature.^11^, ^50^

It appears therefore, that our observation of a different clinical spectrum of South Asian PSP fits within observations of ethnicity‐related differences in PSP, previously noted in other Asian populations. Another consideration to explain the observed differences are environmental factors. However, given that most patients of our cohort lived for decades in the UK, environment is less likely to account for the differences. Further epidemiological and genetic investigations are needed to determine the factors influencing “atypical” presentations.

If the exclusionary criteria of the MDS‐PSP criteria were applied strictly, there would be ambiguity in 10 of our patients on the basis of visual hallucinations (6), limb ataxia (4), autonomic failure (2). When alternative diagnoses were considered, none of our patients fit the MDS‐MSA diagnostic criteria because of the presence of supranuclear gaze palsy. In a study of atypical MSA, the only clinical features that differed in frequencies between a PSP mimic and PSP‐RS/PSP‐P were cognitive domains (any cognitive impairment, frontal lobe dysfunction and memory impairment), being higher in PSP.^51^ Frontal cognitive dysfunction was present in 85% of our patients, making a missed diagnosis of atypical MSA less likely (Table S1). There is a suggestion that sporadic atypical parkinsonism characterized by poor levodopa response, akinetic‐rigidity and early cognitive dysfunction even after the exclusion of patients with clinically probably MSA, PSP and DLB, is more frequent in Afro‐Carribean and Indian populations.^52^ However, gross impairment of vertical saccades is still uncommon in LBD.^6^ Studies to unravel the pathomechanisms of atypical presentations of parkinsonism are much needed, including those of pharmacogenetics. It is likely that we will find overlaps in pathology and phenotype, given the clinical and pathological heterogeneity in neurodegeneration. On the balance, PSP would still be the most probable diagnosis in our cases. Based on current criteria, it might mean that South Asian patients may be underdiagnosed.

Our study has several limitations. Firstly, because of the retrospective nature of the study, the investigations were not uniform across the cohort. Nonetheless, most patients were extensively investigated with imaging, genetic tests, and other relevant diagnostic studies to rule out possible mimics. We also acknowledge that the determination of RBD through clinical history without polysomnographic evidence, allows for non‐REM sleep parasomnias and sleep arousals associated with obstructive sleep apnea to be mistakenly included. *MAPT* haplotype analysis was not available but a recent study of the genetic profile of PSP in an Asian‐Indian population replicated the association of several single nucleotide polymorphisms and in *MAPT* and *STX6*, as well as *H1c* and *H1o MAPT* sub‐haplotypes with PSP.^53^ Secondly, we have no pathological confirmation of our PSP diagnoses. We attempted to overcome this by including only patients who met the probable criteria for PSP which has been found to closely match post‐mortem diagnosis. Pathological studies may be challenging to obtain in South Asian populations due to cultural and religious reasons. A survey of attitudes toward brain donation in Asian patients with PD found that only 50% would consider donating their brains for research with the majority citing emotional discomfort of family members and worry about disfigurement.^54^ Religions that practice burial and those that emphasize on the wholeness of the body after death may be more concerned about the latter reason.^55^ Thirdly, the ethnicity of our patients was based on self‐identification and reporting, rather than being genetically established by haplotype analysis. Finally, as our patients are from a highly specialized movement disorder clinic, there may be a referral bias for more unusual cases of atypical parkinsonism, so that our findings may not be generalizable to the South Asian population. Nevertheless, this is a first‐of‐its‐kind study on South Asian PSP patients that has preliminarily pointed out important differences in the clinical profile compared to cohorts of European descent. While our study did not seek to evaluate differences in disease prevalence, a recent study in the Greater Toronto region found a higher than expected proportion of affected individuals with South Asian ethnic and racial origin,^56^ consolidating the notion that South Asians may have different risk factors or etiological underpinnings for PSP. In addition, a strength of our study is the in‐depth, consistent clinical assessments and records of our patients, providing rich clinical details with video‐documented representative findings.

In conclusion, we present the characteristics of a South Asian cohort of PSP patients with a high percentage of early‐onset disease, family history and atypical clinical manifestations, including cerebellar signs. We highlight that South Asian patients clinically diagnosed with PSP do not fit easily into the PSP phenotypes defined by the current criteria. In addition, ethnicity‐related differences in phenotypes urge long‐term prospective follow‐up studies as well as dedicated neuropathological and genetic studies. Overall, our observations encourage further dissection of the pathogenesis of clinically‐defined PSP, similar to what has occurred in the study of CBS, and the future inclusion of diverse populations in clinical trials.

## Author Roles

(1) Research project: A. Conception, B. Organization, C. Execution; (2) Statistical Analysis: A. Design, B. Execution, C. Review and Critique; (3) Manuscript Preparation: A. Writing of the first draft, B. Review and Critique;

BB: 1A, 1B, 1C, 2C, 3A.

SN: 1A, 1B, 1C, 2A, 2B, 3A.

FM: 2C, 3B.

EM: 3B.

AL: 3B.

MS: 3B.

HRM: 3B.

AB: 3B.

KPB: 1A, 3B.

## Disclosures

**Ethical compliance statement:** Approval by institutional review board was not required for this case report. Written consent was obtained from all patients for the publication of the clinical report and videos in accordance with current ethical standards. We confirm that we have read the Journal's position on issues involved in ethical publication and affirm that this work is consistent with those guidelines.

**Funding Sources and Conflicts of Interest:** No specific funding was received for this work. The authors declare that there are no conflicts of interest relevant to this work.

**Financial Disclosures for the Previous 12 Months:** SN, EM, AL, MS and AB have no disclosures to report. BB has received project funding from the Koetser Foundation and royalties from Oxford University Press. FM is supported by the Edmond J. Safra Foundation. HRM reports paid consultancy from Roche, Aprinoia, AI Therapeutics and Amylyx; lecture fees/honoraria—BMJ, Kyowa Kirin, Movement Disorders Society; research Grants from Parkinson's UK, Cure Parkinson's Trust, PSP Association, Medical Research Council, Michael J. Fox Foundation; co‐applicant on a patent application related to C9ORF72—method for diagnosing a neurodegenerative disease (PCT/GB2012/052140). KPB receives royalties from publication of the Oxford Specialist Handbook *Parkinson's Disease and Other Movement Disorders* (Oxford University Press, 2008), *Marsden's Book of Movement Disorders* (Oxford University Press, 2012), and *Case Studies in Movement Disorders: Common and Uncommon Presentations* (Cambridge University Press, 2017); and has received honoraria/personal compensation for participating as consultant/scientific board member from Ipsen, Allergan, and Merz and honoraria for speaking at meetings from Allergan, Ipsen, Merz, Sun Pharma, Teva, and UCB Pharmaceuticals and from the American Academy of Neurology and the International Parkinson's Disease and Movement Disorders Society.

## Supporting information


**Supplementary Files S1.** Table S1 shows the typical clinical features or clues of progressive supranuclear palsy (PSP) documented throughout disease course, while Table S2 is a list of all the genetic tests that individual cases underwent.

## Data Availability

The data that support the findings of this study are available from the corresponding author upon reasonable request.
