# Supplementary material for: Ethnic Differences in Atypical Parkinsonism—is South Asian PSP Different?
Source: Mov Disord Clin Pract. 2024 Aug 7;11(11):1355–64. doi: 10.1002/mdc3.14182 (PMC11542300; doi:10.1002/mdc3.14182)
Supplement: Supplementary file 1 — Supplementary Files S1. Table S1 shows the typical clinical features or clues of progressive supranuclear palsy (PSP) documented throughout disease course, while Table S2 is a list of all the genetic tests that individual cases underwent. [file MDC3-11-1355-s001.docx]

**Supplementary data**

| **S1. Typical clinical features or clues of PSP documented throughout disease course (*n*=27)** | |
| --- | --- |
| Feature | *n* (%) |
| Vertical supranuclear gaze palsy | 21 (78) |
| Slow vertical saccades | 6 (22) |
| Square wave jerks or eyelid opening apraxia | 13 (48) |
| Falls/ tendency to fall on pull test within 3 years | 19 (70) |
| Progressive gait freezing within 3 years | 3 (11) |
| Parkinsonism (axial predominance and levodopa-resistant) | 6 (22) |
| Parkinsonism (asymmetric) | 17 (63) |
| Non-fluent/ agrammatic primary progressive aphasia | 2 (7) |
| Frontal cognitive dysfunction (bradyphrenia/ dysexecutive syndrome/ reduced verbal fluency) | 23 (85) |
| Perseveration (applause/ echolalia/ palilalia) | 17 (63) |
| Limb apraxia | 14 (52) |
| Limb dystonia | 16 (59) |
| Limb myoclonus | 5 (19) |
| Dysarthria | 24 (89) |
| Dysphagia | 17 (63) |

| **S2. Table of clinical features and investigations of individual patients** | | | | | |
| --- | --- | --- | --- | --- | --- |
| ID | Core features | Supportive clinical clues | Atypical features | Neuroimaging | Other investigations |
| 1 | O1+A3 | CC2 | EOPSP, RBD, abnormal posture, cerebellar, FHx | Frontoparietal atrophy | CSF, IgLON5, genetics |
| 2 | O1+P1 | CC1/2/3 | EOPSP, RBD, cerebellar | Midbrain and cerebellar atrophy | Genetics, oxysterol |
| 3 | O1+P1 | CC1/2/3 | Cerebellar | SCP and cerebellar atrophy | Genetics |
| 4 | O1+P1 | CC1/2/3 | EOPSP, LID, FHx | Midbrain atrophy | CSF, genetics |
| 5 | O1+P1 | CC2/3 | LID | Biparietal atrophy | CSF, genetics |
| 6 | O1+A1 | CC1/2/3 | RBD, cerebellar | Generalized atrophy | - |
| 7 | O2+P1 | CC1/2/3 | FHx | Midbrain atrophy | Genetics |
| 8 | O1+A3 | CC1/2/3 | Abnormal posture, dysautonomia | Generalized atrophy | - |
| 9 | O2+P1 | CC1/2 | RBD, VH dysautonomia, cerebellar, FHx | Pontine and cerebellar atrophy, putaminal signal change | CSF, genetics |
| 10 | O1+A1 | CC1/2 | VH | - | - |
| 11 | O2+C2 | CC1/2/3 | FHx | Midbrain atrophy | Genetics |
| 12 | O1+P1 | CC1/2/3 | EOPSP, cerebellar | Midbrain atrophy | CSF, genetics |
| 13 | O1+P1 | CC1/2/3 | Cerebellar | Midbrain and cerebellar atrophy | CSF |
| 14 | O1+P1 | CC1/2/3 | RBD, VH, abnormal posture | Midbrain atrophy | CSF, IgLON5, genetics |
| 15 | O1+P1 | CC1/2 | RBD | Generalized atrophy | IgLON5, genetics |
| 16 | O2+A1 | CC1 | - | Normal | CSF |
| 17 | O1+P1 | CC1/2/3 | - | Midbrain atrophy | Genetics |
| 18 | O1+A3 | CC1/2/3 | EOPSP | Frontoparietal atrophy | CSF, genetics |
| 19 | O1+P1 | CC1/2 | RBD, VH | Midbrain atrophy | CSF, genetics |
| 20 | O1+P1 | - | Cerebellar | Frontal atrophy | CSF, genetics |
| 21 | O1+P1 | CC2/3 | RBD, VH | Midbrain atrophy | Genetics |
| 22 | O1+P1 | CC2 | RBD | Normal | Genetics |
| 23 | O1+A1 | CC1/2/3 | - | Generalized atrophy | CSF, IgLON5 |
| 24 | O1+P1 | CC2/3 | EOPSP, RBD, VH, cerebellar | Midbrain atrophy | - |
| 25 | O1+A3 | CC2 | RBD, LID | Generalized atrophy | - |
| 26 | O2+P1 | CC1 | - | Midbrain atrophy | Amyloid PET, IgLON5 |
| 27 | O1+P1 | CC1/2/3 | - | Generalized atrophy | IgLON5 |

O – ocular motor dysfunction; P – postural instability; A – akinesia; C – cognitive dysfunction; CC – clinical clues; CSF – cerebrospinal fluid (analysis for neurodegenerative biomarkers including Ab42, Aβ40/Aβ42 ratio, total tau and phosphorylated tau); EOPSP – early-onset progressive supranuclear palsy; FHx – (positive) family history; LID – levodopa-induced dyskinesia; PET – positron emission tomography; RBD – rapid eye movement behavior disorder; SCP- superior cerebellar peduncle; VH – visual hallucination

| **S2. Genetic tests performed (*n=17*)** | | | | |
| --- | --- | --- | --- | --- |
| ID | Tests | FHx | EO-PSP | Cerebellar signs |
| 1 | Dementia panel, *C9orf72, DCTN1*, *POLG, ATX2/3/7* | + | + | + |
| 2 | SCA panel, *PPP2R2B, TBP, ATN1,* *POLG,* mtDNA large scale rearrangement, *C9orf72*, dementia panel | - | + | + |
| 3 | Dementia panel, *C9orf72, ATX1/2/3/7* | - | - | + |
| 4 | WGS – parkinsonism panel; dementia panel, mitochondrial genome, mtDNA maintenance gene panel | + | + | - |
| 5 | Dementia panel, *C9orf72* | - | - | - |
| 7 | Dementia panel, YOPD panel, mtDNA maintenance gene panel | + | - | n/a |
| 9 | SCA panel, *TBP, C9orf72, POLG, PEO1* | + | - | + |
| 11 | WGS – parkinsonism panel; mitochondrial genome | + | - | - |
| 12 | *JPH3, HTT, C9orf72*, *ATN1,* *DCTN1, POLG,* MELAS, ATX2/3/7 | - | + | + |
| 14 | WGS – adult-onset neurodegeneration panel | - | - | - |
| 15 | SCA panel, *PPP2R2B, TBP, POLG* | - | - | - |
| 17 | Dementia panel, *ATXN1/2/3, C9orf72, POLG, PEO1* | - | - | - |
| 18 | Dementia panel, *C9orf72* | - | + | - |
| 19 | Dementia panel, *C9orf72, PRNP, POLG, MT-AT6* | - | - | - |
| 20 | *C9orf72, MAPT, GRN, DCTN1, ATX1/2/3/7* | - | - | + |
| 21 | *ATXN2* | - | - | - |
| 22 | *MAPT* | - | - | n/a |

mtDNA: mitochondrial deoxyribonucleic acid, SCA: spinocerebellar ataxia, WGS: whole genome sequence, YOPD: young-onset Parkinson disease

- Dementia panel consists of *APP, CHMP2B, CSF1R, DNMT1, FUS, GRN, HTRA1, ITM2B, MAPT, NOTCH3, PRNP, PSEN1, PSEN2, TARDBP, TREM2, TYROBP, VCP*
- SCA panel consists of *ATXN1, ATXN2, ATXN3, CACNA1A, ATXN7*
- YOPD panel consists of *PARK2, PARK7, PINK1, SNCA, VPS35, FBXO7*
- WGS parkinsonism panel consists of *PRKN, ATP13A2, ATP1A3, C19orf12, CSF1R, DCTN1, DNAJC6, FBXO7, FTL, GCH1, GRN, LRRK2, MAPT, OPA3, PANK2, PARK7, PINK1, PLA2G6, PRKRA, RAB39B, SLC30A10, SLC39A14, SLC6A3, SNCA, SPG11, SPR, SYNJ1, TH, TUBB4A, VPS13A, VPS35, WDR45, CHCHD2, GBA, TAF1, ANO3, ATXN2, ATXN3, C9orf72, EIF4G1, GIGYF2, GNAL, HTRA2, HTT, IPPK, JPH3, NR4A2, SGCE, SLC41A1, SNCAIP, TBP, THAP1, TOR1A, UCHL1, EPHB4*
- WGS adult-onset neurodegeneration panel v4.34 can be found at: https://panelapp.genomicsengland.co.uk/panels/474/
